# Thermoelectric Properties of Tetrahedrites Produced from Mixtures of Natural and Synthetic Materials

**DOI:** 10.3390/ma18061375

**Published:** 2025-03-20

**Authors:** Beatriz A. Santos, Luís Esperto, Isabel Figueira, João Mascarenhas, Elsa B. Lopes, Rute Salgueiro, Teresa P. Silva, José B. Correia, Daniel de Oliveira, António P. Gonçalves, Filipe Neves

**Affiliations:** 1 C2TN—Center for Nuclear Sciences and Technologies, Department of Nuclear Sciences and Engineering, Instituto Superior Técnico, University of Lisbon, 2695-066 Bobadela-LRS, Portugal; beatriz.santos@ctn.tecnico.ulisboa.pt (B.A.S.); eblopes@ctn.tecnico.ulisboa.pt (E.B.L.); 2 LNEG, Laboratório Nacional de Energia e Geologia, Estrada do Paço do Lumiar, 22, 1649-038 Lisboa, Portugal; luis.esperto@lneg.pt (L.E.); isabel.figueira@lneg.pt (I.F.); joao.mascarenhas@lneg.pt (J.M.); brito.correia@lneg.pt (J.B.C.); 3 LNEG, Laboratório Nacional de Energia e Geologia, Estrada da Portela, Bairro do Zambujal–Alfragide, Apartado 7586, 2610-999 Amadora, Portugal; rute.salgueiro@lneg.pt (R.S.); teresa.pena@lneg.pt (T.P.S.); daniel.oliveira@lneg.pt (D.d.O.)

**Keywords:** tetrahedrite–tennantite series, mechanochemical synthesis, Seebeck coefficient, electrical resistivity, power factor

## Abstract

Thermoelectric materials have considerable potential in the mitigation of the global energy crisis, through their ability to convert heat into electricity. This study aims to valorize natural resources, and potentially reduce production costs, by incorporating tetrahedrite–tennantite (td) ores from the Portuguese Iberian Pyrite Belt into synthetic samples. The ore samples were collected in a mine waste at Barrigão and as “dirty-copper” pockets of ore from the Neves Corvo mine. Subsequently, high-energy ball milling and hot pressing were employed in the production of thermoelectric materials. These are characterized by XRD, SEM/EDS, and thermoelectrical properties. The complete dissolution of the dump material sulfides with the synthetic tetrahedrite constituents led to an increase in the amount of the tetrahedrite–tennantite phase, which was made up of a tetrahedrite–tennantite–(Fe) solid solution. The thermoelectric characterization of these materials is provided, revealing that most of the combined synthetic ore samples displayed better results than the pristine tetrahedrite, mostly due to higher Seebeck coefficient values. Furthermore, the best thermoelectric performance is achieved with 10% of ore, where a power factor of 268 µW.K^−2^.m^−1^ is reached at room temperature.

## 1. Introduction

The search for new, reliable, low-cost, and eco-friendly energy sources is continually growing as our energy consumption increases. Therefore, thermoelectric (TE) materials have been increasingly sought after in recent decades due to their reliable applicability, mainly in waste heat recovery, refrigeration, and electrical power generators, given their ability to directly convert heat into electricity and vice-versa [1,2,3].

Despite their attractive properties and wide range of applications [4], these materials present some adverse problems, namely their low efficiency. Most TE materials have maximum efficiency values close to 5% [5], with those that perform best containing rare or toxic elements. Consequently, the need to find and develop improved and harmless TE materials has increased over the years along with the growing demand for new, powerful, and environmentally friendly energy sources [5,6,7].

The conversion of thermal energy into electrical energy, and vice-versa, is explained by the Seebeck and Peltier effects, respectively, while the Thomson effect describes the presence of an electromotive force throughout a TE material whenever a temperature gradient and charge current are applied [1,8].

The performance of a material for TEs might be expressed by its figure of merit, zT = S^2^T/ρκ, which is related to the absolute temperature (T), Seebeck coefficient (S), electrical resistivity (ρ), and thermal conductivity (κ) of the material. The thermal has two main contributions, the lattice thermal conductivity, k_l_, and electron thermal conductivity, k_e_ [1,9,10].

One of the biggest challenges when working with thermoelectric materials is the simultaneous optimization of all the mentioned parameters. The electrical and thermal conductivity (the electronic contribution part) are directly related to the electrons’ movement and hence to each other. The Seebeck coefficient, on the other hand, benefits from a lower charge carrier density and higher band gaps, which means by improving the Seebeck coefficient tendentially, the electric conductivity will diminish. In this sense, careful tuning of the thermoelectric parameters is required in order to achieve better performance.

**Figure 1:**
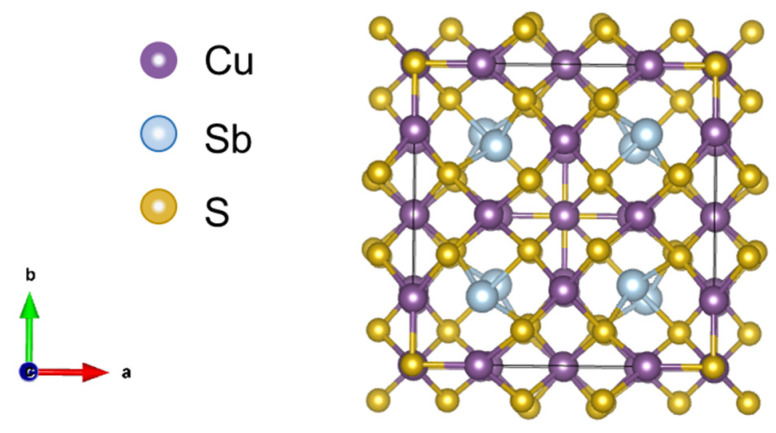
Schematic representation of a pristine tetrahedrite crystal structure.

Conventional high-performance TE materials contain expensive, rare, or toxic elements, such as tellurium or lead. As an alternative to them, tetrahedrites (Cu_12_Sb_4_S_13_) have been a focus of attention in the field of TE materials due to their abundance on Earth, low cost, low toxicity, low price, and reduced intrinsic thermal conductivity [6,11]. They belong to the sulfosalt minerals, presenting a complex crystal structure, with 58 atoms assembled in a body-centered cubic (bcc) unit cell, with the space group $I4\bar{3}m$ [11], as it is represented in Figure 1. When found naturally in the ores, it typically has iron or zinc, substitutions on some of the copper sites, as well as arsenic instead of the antimony. When the latter is applied, it is designated as tennantite (Cu_12_As_4_S_13_), and it has the same crystal structure as the tetrahedrite.

**Figure 2:**
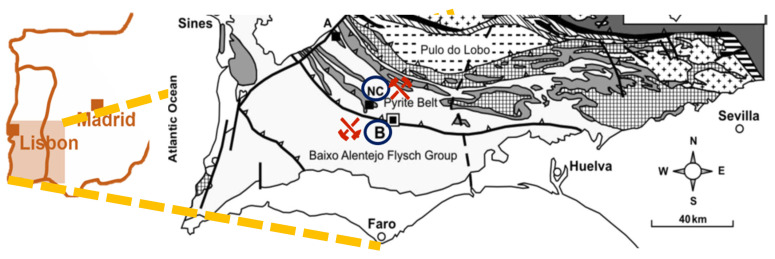
Map of the region of interest in the Iberian Pyrite Belt, with the Neves Corvo and Barrigão mines highlighted.

The potential of using naturally occurring tetrahedrites has already been explored in thermoelectric applications, and some studies [12,13,14] have reported their competitive properties [15,16]. In this work, a combination of synthetic tetrahedrites and mineral tetrahedrites was tested, the latter coming from two sources, the dumps of the Barrigão mine and the Neves Corvo mine, both located in the Iberian Pyrite Belt (IPB), as exhibited in Figure 2 [17]. Thus, the aim of this study was to find a more environmentally friendly alternative to the commonly employed thermoelectric materials for low/middle-range temperature applications and add value to natural resources. To achieve the proposed goal, mixtures of different mass proportions (from 10% to 50%) of tetrahedrite–tennantite ore with synthetic tetrahedrite were tested in order to find the most suitable one. To produce these samples, a two-step powder technology sequence was employed, starting with mechanochemical synthesis followed by hot pressing. Additionally, heat treatment was employed with the intent of homogenizing the main phase, thus enhancing the TE properties. The thermoelectric performance of these samples was evaluated depending on the ore content and the influence of the location where the natural minerals were collected.

## 2. Materials and Methods

### 2.1. Material Preparation

The production of tetrahedrite-based materials included the following steps:

1. Tetrahedrite–tennantite ores in two distinct settings in the IPB were sampled: at the Barrigão mine, surface samples were collected from the widespread dumps, while at Neves Corvo, the ore samples were collected underground from one of the intersected “dirty-copper” pockets of ore. The Barrigão ore (BO) samples consist of tetrahedrite–tennantite containing Fe and richer in As than in Sb, coexisting with chalcopyrite (CuFeS_2_) and quartz (SiO_2_), and can be generically considered as As-rich tetrahedrite ore [17,18,19]. The Neves Corvo ore (NCO) shows a more heterogeneous assemblage composed predominantly of tennantite (also incorporating Fe) with dispersed grains of pyrite (FeS_2_) and minor zones of chalcopyrite, stannite (Cu_2_FeSnS_4_), quartz, and galena (PbS) minerals [19]. Consequently, the Neves Corvo can be designated as tennantite-(Fe) ore. Full details on ore preparation and their characterization are given in [16,19].

2. Synthetic Cu_12_Sb_4_S_13_ tetrahedrites (syn-td) were produced by mechanochemical synthesis (MCS) from mixtures of commercial elemental powders of copper (Cu; Alfa Aesar (Haverhill, MA, USA), antimony (Sb; Sigma-Aldrich (affiliated of Merck KGaA, Darmstadt, Germany)), and sulfur (S; Alfa Aesar (Haverhill, MA, USA)). Multiphase compounds, consisting mainly of tetrahedrite and famatinite phases, were formed with the MCS process [19]. Detailed information regarding the materials and experimental conditions followed on this step was previously reported [19].

3. “Ore/syn-td” powder mixtures (hereinafter referred to as BO/syn-td and NCO/syn-td, for mixtures with Barrigão and Neves Corvo ores, respectively) were produced by MCS, containing different mass ratios of ore (10% to 50%). The MCS was performed in a high-energy ball milling PM 400 from Retsch (Retsch GmbH, Haan, Germany), at 380 rpm for 2 h, with a ball-to-powder ratio of 20:1 and without any additional fluid medium. More details concerning the MCS experimental conditions can be found in [19]. At the end of this process, it was observed that the main constituent was a tetrahedrite–tennantite–(Fe) phase ((Cu,Fe)_12_(Sb,As)_4_S_13_) coexisting with other minor secondary phases, namely quartz and famatinite (Cu_3_SbS_4_), in the BO/syn-td samples, and quartz, pyrite, famatinite, and luzonite (Cu_3_AsS_4_) in the NCO/syn-td [19].

4. Densification was made by hot pressing the MCS powders prepared in steps 2 and 3. A Termolab SV-Prensa 200/2018 (Termolab, Portugal) uniaxial hot press (HP) and a high-density graphite mold were used for this step. The temperature and pressure were raised to 575 °C, at a rate of 25 °C.min^−1^, and 43.7 MPa, and maintained under these conditions for 1.5 h in an inert atmosphere (Ar). Pellets of 10 mm in diameter and 1 mm in height were obtained.

5. Homogenization heat treatment (HT) of the hot-pressed pellets at 415 °C for 6 days in vacuum (10^−2^ mbar) was performed. This step was performed only in the syn-td and BO/syn-td (10/90% and 20/80%) samples.

### 2.2. Characterization Methods

Powder X-ray diffraction (XRD) measurements were performed on a Bruker diffractometer D2 Phaser (from Billerica, MA, USA), with Bragg–Brentano geometry and Cu Kα radiation, applying 45 kV of tension and 35 mA of current. The XRD data were recorded in a 2θ range of 10° to 70°, at a scan speed of 1 s/step with a 0.02 step. Phase identification was made through a comparison of the collected diffractograms with reference patterns taken from the Crystallography Open Database (COD) [20] using Origin Lab (Orgin Pro 9.0) and Powder Cell software (version 2.4). The microstructure of the processed materials was assessed by scanning electron microscopy (SEM) using a Philips XL30 field emission SEM (FEI, Eindhoven, The Netherlands), equipped with a backscattered electron detector (BSE), and a Thermo Scientific™ UltraDry energy dispersive X-ray spectroscopy (EDS) detector (Thermo Fisher Scientific, Waltham, MA, USA) to evaluate the phase composition.

The Seebeck coefficient (S) was determined by employing an adaptation of the technique developed by Chaikin for measuring organic single crystals and performed in a closed-cycle cryostat from 30 to 300 K [21]. For measuring the electrical resistivity (ρ) of the samples, the “Four points method” was employed with an AC resistance bridge device, the Linear Research LR-700 [22]. The amplitude of the current imposed during the measurements was I = 1 mA.

## 3. Results and Discussion

### 3.1. Structural and Microstructural Characterization

**Figure 3:**
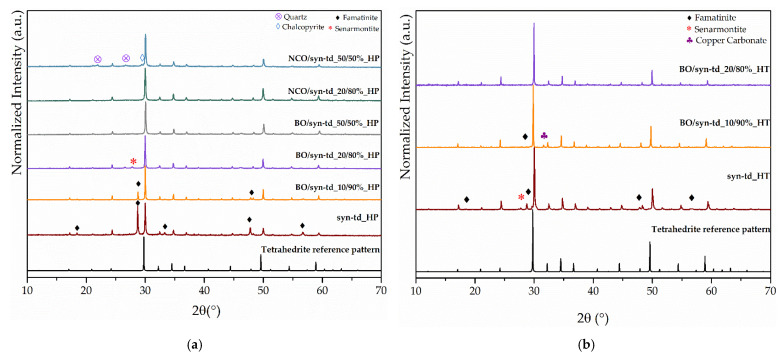
Typical XRD patterns (**a**) of hot-pressed (HP) samples and (**b**) heat-treated (HT) samples (the black XRD pattern at the bottom of each figure corresponds to the reference tetrahedrite pattern obtained from the database file COD ID #9004148 [20,24]).

Typical X-ray diffractograms (XRDs) for the hot-pressed (HP) samples are shown in Figure 3a. For all ore/syn-td samples, the main XRD peaks are sharp and well defined, and can be indexed to the tetrahedrite–tennantite–(Fe) phase. Additionally, a slight shift towards higher angles of these XRD peaks is evident when compared to the 2Ө positions of the tetrahedrite reference pattern (shown at the bottom of the figure). This shift results from the reduction of the lattice parameter due to the replacement of Sb by As. Moreover, samples with high concentrations of NCO exhibit quartz and chalcopyrite secondary phases, as identified by XRD. This may be associated with the location of the ore mines, which ultimately defines the composition of the ores. Concerning other phases present in the samples, the XRD pattern reveals the coexistence of tetrahedrite and famatinite, which has already been reported in previous studies made on synthetic materials [23], indicating the difficulty in obtaining pure tetrahedrite. The mineral composition of the initial ores can be found in the Supplementary Informatio (S.I.).

**Figure 4:**
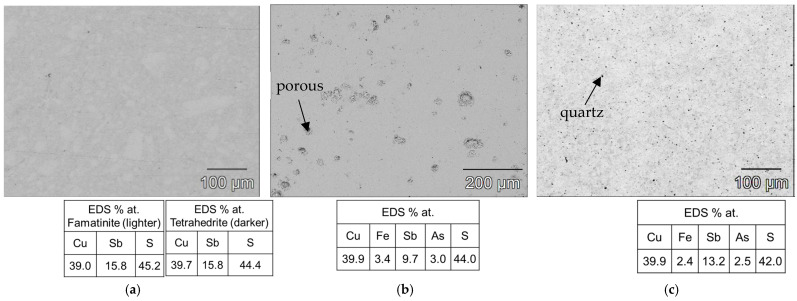
Typical SEM/BSE images of the hot-pressed samples with their respective atomic concentration (%) for the matrix through EDS, here exemplified for the (**a**) syn-td_HP, which exhibits a strong presence of both tetrahedrite and famatinite, (**b**) BO/syn-td_10/90%, and (**c**) BO/syn-td_20/80%.

**Figure 5:**
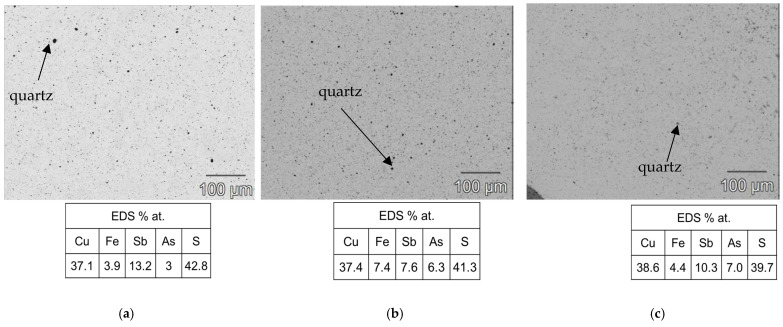
Typical SEM/BSE images of the hot-pressed samples with their respective atomic concentration (%) for the matrix through EDS, here exemplified for the (**a**) NCO/syn-td_20/80%, (**b**) NCO/syn-td_50/50%, and (**c**) BO/syn-td_50/50%.

**Figure 6:**
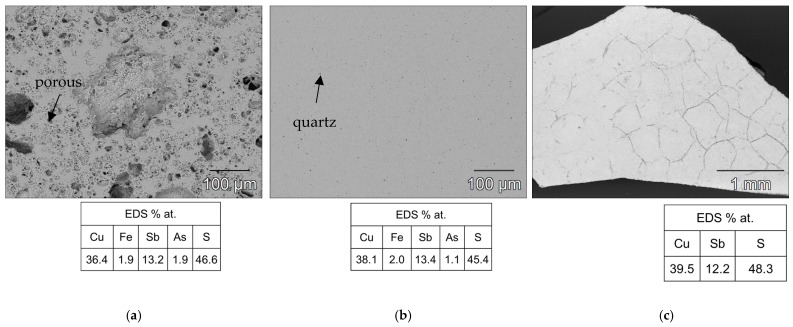
Typical SEM/BSE images of the thermally treated samples with their respective atomic concentration (%) for the matrix through EDS. (**a**) BO/syn-td_10/90%_HT, (**b**) BO/syn-td_20/80%_HT, and (**c**) syn-td_HT.

As shown in Figure 3b, the heat treatment highlighted the structural stability of the tetrahedrite–tennantite–(Fe) phase. Some secondary phases were eliminated by the heat treatment, while others noted a significant reduction in the XRD peak intensity of, more specifically, famatinite, which is clearly observed in the syn-td_HT and BO/syn-td_10/90%_HT samples. The latter completely stabilized the tetrahedrite–tennantite–(Fe) phase, eliminating the presence of famatinite. Additionally, no extra XRD peaks, besides those corresponding to the tetrahedrite–tennantite–(Fe) phase, were indexed in the BO/syn-td_20/80%_HT. These analyses demonstrated the unchangeable crystal structure of the samples, and registered only the reduction of the secondary phases, further enhancing their main phase (tetrahedrite) stabilization. Typical SEM/BSE images of the HP samples are presented in Figure 4, Figure 5 and Figure 6, exhibiting a high homogeneity throughout the materials, correlating well with the XRD results shown in Figure 3a. Higher quartz and other secondary phase content is observed in the NCO/syn-td_50/50% sample when compared to the BO/syn-td_50/50% one, with the black spots in the SEM/BSE images corresponding to micro-inclusions of quartz in the tetrahedrite–tennantite–(Fe) matrix. The syn-td_HP sample appears to be differently grey-shaded, consistent with the presence of the tetrahedrite–tennantite–(Fe) and famatinite phases (lighter grey shade). Additionally, observations confirm the absence of porosity, except for sample BO/syn-td_10/90%, which formed inclusions and revealed a high porosity, indicating that this proportion of synthetic with natural ore was not adequate to form a compact sample.

Figure 5 exhibits typical SEM/BSE images of (a) BO/syn-td_10/90%_HT, (b) BO/syn-td_20/80%_HT, and (c) syn-td_HT samples (after heat treatment), showing that lower ore content leads to a higher degradation of the materials, increasing their brittleness: in the case of the pristine synthetic sample, despite almost eliminating the presence of famatinite, it formed significant cracks throughout the material, breaking when maneuvered. A similar case occurred for the BO/syn-td_10/90%_HT sample, which had its famatinite phase almost eradicated, but its original porosity was noticeably aggravated. Notwithstanding, sample BO/syn-td_20/80%_HT was a remarkable exception, revealing no other phases besides the intended tetrahedrite as shown previously in the XRD diffractogram (Figure 3b) and did not present signs of suffering cracks or pores.

### 3.2. Thermoelectric Properties

**Figure 7:**
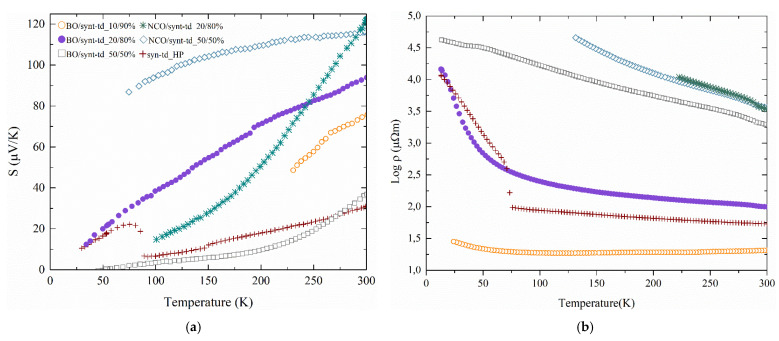
Thermoelectric properties of the hot-pressed samples: (**a**) Seebeck coefficient and (**b**) electrical resistivity.

The Seebeck coefficient (S) and the electrical resistivity (ρ) of representative HP samples are shown in Figure 7. Synthetic doped tetrahedrites are highly doped degenerate semiconductors that present a semiconducting behavior in resistivity and metallic-like behavior in the Seebeck due to the high degeneracy present in the band structure. The highest Seebeck coefficients at room temperature (300 K) were obtained for the NCO/syn-td_20/80% and NCO/syn-td_50/50% samples, with values of 121 μV.K^−1^ and 116 μV.K^−1^, respectively, which agreed well with the higher activation energies and more resistive values observed in the resistivity curves. For the Barrigão ore samples, the BO/syn-td_20/80% displayed the highest Seebeck of 90 μV.K^−1^ (300 K), which is similar to those previously described [11,25,26]. This can be correlated to the presence of secondary phases, which were reported to be mostly quartz and chalcopyrite for NCO ore, senarmontite for BO ore, and famatinite for high synthetic composition samples, respectively, which can interfere with the band structure, acting as composites, hence affecting the density of states and consequently the Seebeck coefficient values [27]. Depending on the nature of the secondary phases present in the sample, they might lead to an increase in the density of states. If the newly added states are close to the Fermi level, they can increase the slope of the band, which leads to an enhancement of the Seebeck coefficient [28]. However, this effect might be reversed if the charge carrier concentration strongly increases, since for semiconductors, the Seebeck coefficient is inversely proportional to the charge carrier concentration [29].

Both phenomena were observed in these samples. For sample syn-td_HP, a higher content of famatinite was observed coupled with the lowest exhibited value of Seebeck coefficient, which is concordant with what is described in the literature [26] and with the previous statement, since it is highly likely that famatinite suffers from a bipolar effect in its charge carriers due to its low band gap (0.27 eV [30]). Since it has a narrow band gap at ordinary temperatures, it is expected that more carriers become thermally excited at the same temperature, hence a lower average energy per charge carrier, ergo reducing the Seebeck coefficient [31]. Contrastingly, the tetrahedrite has a larger band gap of 1.4 eV [32], thus possessing a lower number of intrinsic charge carriers, favoring a higher Seebeck coefficient. Nevertheless, with the high presence of famatinite in the syn-td_HP sample, the Seebeck coefficient becomes highly influenced by it and decreases its value when compared with the other samples, particularly with BO/syn-td_10/90% (which is the closest to the composition of syn-td), which showed a value of 75 μV.K^−1^ (at 300 K).

All the NCO/syn-td samples and Bo/syn-td_20/80% showed an opposite effect to the syn-td, where the presence of quartz as a secondary positively influences the Seebeck coefficient, possibly due to the high band gap of quartz, ~9.65 eV [33]. This effect was mostly noticed in the NCO ore samples, which displayed a higher content of quartz in their phase composition. Since quartz is an insulator, it can act as an energy filtering barrier (particularly when present in nanoscale), limiting and scattering the low energy charge carriers' concentration [34], thus improving the Seebeck coefficient of these samples. Likewise, senarmontite might act as an energy filtering barrier, creating interfaces in a similar fashion as quartz, as it has been reported in the literature for other semiconductors [35]. The presence of chalcopyrite would possibly reduce the Seebeck coefficient, since its charge carriers (n-type) [36] are of a different type than tetrahedrite (p-type), creating a bipolar effect, but, due to the larger presence of quartz, this effect was mitigated.

Concerning the electrical resistivity (ρ), all the aforementioned effects that aided or harmed the Seebeck coefficient are expected to display an opposite effect in the electrical resistivity, unless when properly engineered. For this work, the presence of most secondary phases was inherited from the nature of the ores themselves, having no intended manipulation. Higher ore content leads to brittle samples, consequently increasing the porosity of the samples, which, aligned with the noticeable presence of quartz, greatly amplifies the electrical resistivity.

The syn-td_HP exhibits the expected metal–insulator transition at 85 K, also seen in the Seebeck curve, although the resistivity values are one order of magnitude higher than those reported by Suekini et al. [37]; this could be due to the different preparation methods used and presence the of famatinite in our samples.

The lowest resistivity value was obtained for the BO/syn-td_10/90% sample (21 μΩ.m at 300 K). The NCO/syn-td samples, which showed the highest Seebeck coefficients, had very high electrical resistivities, 3412 and 3628 μΩ.m at 300 K for NCO/syn-td_20/80% and NCO/syn-td_50/50%, respectively, which correspond to the high activation energies observed in the resistivity versus temperature curves, making them unsuitable for TE applications. These high activation energies were also observed in Bo/syn-td_50/50%, which revealed poor thermoelectric performance in all the measured properties. In this regard, sample BO/syn-td_10/90%, followed by BO/syn-td_20/80%, displayed the best overall performance thus far, considering that it managed to acquire the best equilibrium between the analyzed thermoelectric properties.

For higher temperatures, the performance of the best materials is expected to improve significantly as the Seebeck should increase in a tendential linear way and the resistivity should decrease due to the semiconducting behavior.

**Figure 8:**
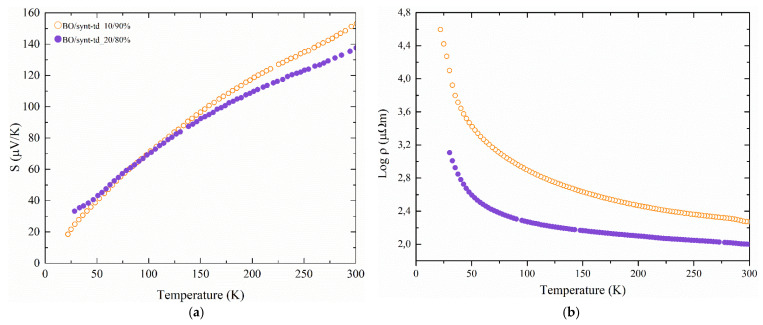
Thermoelectric properties of the heat-treated samples: (**a**) Seebeck coefficient and (**b**) electrical resistivity.

Regrettably, the effect of the heat treatment did not correspond to its intended purpose, which was to enhance the homogeneity and thermoelectric properties. In fact, it led to a general degradation of the samples, hence greatly decreasing their mechanical endurance and worsening their electrical resistivity due to the higher porosity (Figure 6a,c); such is the case for the BO/syn-td_10/90%_HT, which registered a significant increase from 21 to 186 μΩ.m at 300 K, as it is exhibited in Figure 8, where Seebeck coefficient and electrical resistivity values for the heat treated samples are showed. However, as presented in Figure 6b and Figure 8, the heat treatment positively influenced the BO/syn-td_20/80%_HT sample, which exhibited better performance after the HT, keeping its original electrical resistivity value and improving the Seebeck coefficient.

The determination of the Seebeck coefficient (S) and electrical resistivity (ρ) allowed for the calculation of the power factor (PF = S^2^/ρ). These results are displayed in Table 1.

The best power factor values were obtained for the BO/syn-td_10/90% samples with a value of 268 μW.K^−2^.m^−1^. These results show a comparable power factor value for 10% and 20% BO ore content (particularly BO/syn-td_10/90% and BO/syn-td_20/80%_HT samples), exhibiting values in the same order of magnitude when compared to optimized synthetic pristine tetrahedrite reported in the literature [23].

Compared with other reports in the literature where natural mineral tetrahedrites are used, the work of Levinský [10,14] is highlighted here as the research group exhibited the importance of the ore collecting site, which affects its composition and therefore the thermoelectric properties. In their work, most of the mixtures employed were in the ratio of 50:50 with synthetic Ni-content tetrahedrite (monophasic) exhibiting power factor values between 80 and 200 μW.K^−2^.m^−1^ at 300 K, which are similar to the ones reported in this article. Lu and Morelli’s work [15] achieved a maximum power factor value of ~225 μW.K^−2^.m^−1^ at 300 K, with a 50:50 mixture as well; however, most of the results displayed values lower than 50 μW.K^−2^.m^−1^ (300 K). In both reported works, what diminished their performance was the electrical resistivity, which can be tuned with proper compaction and sintering condition optimization and composition.

It is interesting to perceive that despite the variability of the natural ore composition and secondary phase contents, which may harm the thermoelectric performance, this issue may be overcome by the right production conditions and finding the enhanced tuning for the respective collecting site, as it may be observed by the final results acquired in all these works, regardless of their initial composition or synthesis (and sintering) routes.

Considering that the results presented in Table 1 were measured at room temperature, it is expected that these samples will perform better at higher temperatures, up to 623 K, which is the maximum working temperature for tetrahedrites, after which the formation of oxides and thermal degradation of tetrahedrites start to occur for most compositions [38,39,40,41].

Overall, the results are promising and encouraging, confirming the possible direct usage of ore samples and dump material as a source of raw materials for the synthesis of tetrahedrite-based materials for TE applications with all the potential environmental/economic gains that can be obtained.

## 4. Conclusions

In this work, successful combinations of synthetic tetrahedrite with natural ores, collected in the Portuguese zone of the Iberian Pyrite Belt (Neves Corvo and Barrigão mines), were accomplished via mechanochemical synthesis and hot pressing. These samples were tested for thermoelectric applications.

Samples combined with Neves Corvo ore had higher quartz and pyrite content, which give worse overall thermoelectric performance due to the high electrical resistivity. In contrast, samples with ore from the Barrigão mine displayed promising results, particularly the samples containing 10 and 20% (BO/syn-td_10/90% and BO/syn-td_20/80%), which exhibited the best equilibrium between achieving a high Seebeck coefficient and keeping a low electrical resistivity.

Thermal treatment at 415 °C for 6 days was performed in order to stabilize the tetrahedrite phase, eliminating the presence of other secondary ones, with the intent to optimize the thermoelectric performance. Despite this endeavor, the results were not the expected ones and led to the mechanical degradation of most of the tested samples. Nevertheless, sample BO/syn-td_20/80%_HT greatly improved its properties.

Overall, the best sample was the BO/syn-td_10/90%, which reached a power factor of 268 μW.K^−2^.m^−1^ at 300 K. This work shows that with the right tuning of natural ore and synthetic tetrahedrite, better results than the reported literature can be obtained than merely synthetic tetrahedrites, creating a valorization of natural resources that would otherwise be considered waste.

## Figures and Tables

**Table 1:** Thermoelectric properties: electrical resistivity (ρ), Seebeck coefficient (*S*), and power factor (PF) at room temperature (300 K) for the hot-pressed and heat-treated samples.

Sample ID	ρ (μΩ.m)	*S* (μV.K^−1^)	PF (μW.K^−2^.m^−1^)
syn-td_HP	53.6	31	18
BO/syn-td_10/90%	21	75	268
BO/syn-td_20/80%	99	90	84
BO/syn-td_10/90%_HT	33	96	125
BO/syn-td_20/80%_HT	84	137	190
BO/syn-td_50%50	1929	37	0.7
NCO/syn-td_20/80%	3412	121	4
NCO/syn-td_50/50%	3628	116	3.7

## Data Availability

The original contributions presented in this study are included in the article/Supplementary Materials. Further inquiries can be directed to the corresponding author.

## Supplementary Materials

The following supporting information can be downloaded at: https://www.mdpi.com/article/10.3390/ma18061375/s1.
