# Supplementary material for: Thermoelectric Properties of Tetrahedrites Produced from Mixtures of Natural and Synthetic Materials
Source: Materials (Basel). 2025 Mar 20;18(6):1375. doi: 10.3390/ma18061375 (PMC11944066; doi:10.3390/ma18061375)
Supplement: Supplementary file 1 [file materials-18-01375-s001.zip › materials-3451009-supplementary.pdf]

Supplementary Information

Table S1 Mineral analysis of the Barrigão ore used in the samples in comparison with the literature from Reiser et al. All samples analysed at Actlabs, Ontario, Canada.

**Analyte symbol**	**Unit**	***Reiser (2011) average***			**Barrigão (July 2019)**
	symbol	Mean	Max.	Min.	
**Cu**	%	*15.89*	27.5	4.18	> 1
**S**	%	*10.53*	>20	3.03	14.00
**Fe**	%	*7.79*	16.4	1.69	11.50
**Al**	%	*1.92*	7.28	0.95	2.94
**As**	%	*1.72*	5.95	0.06	1.21
**Sb**	%	*0.72*	1.76	0.05	0.39
**Ca**	%	*0.54*	2.92	0.11	1.57
**Mg**	%	*0.24*	1.4	0.05	1.00
**P**	%	*0.12*	0.22	0.05	0.17
**Zn**	ppm	*820.84*	5500	76	880.00
**Bi**	ppm	*253.47*	1070	44.9	92.00
**Sn**	ppm	*192.65*	872	16	199.00
**Ge**	ppm	*30.12*	280	3.9	28.40
**Pb**	ppm	*15.64*	120	3	22.00
**Ag**	ppm	*54.66*	188	5	45.10
**In**	ppm	*2.72*	7	1	2.10

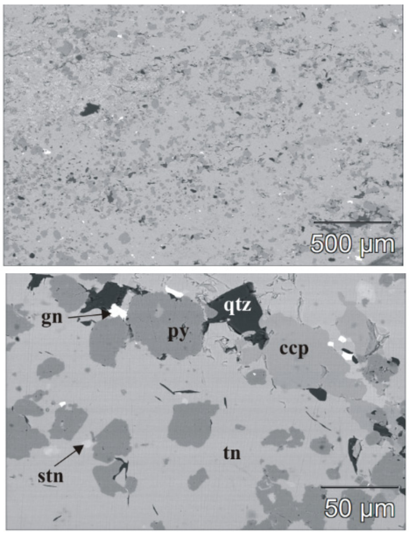

	Cu	Fe	Sb	As	S	Sn	Possible mineral formula
Neves Corvo							
tn	30.4±0.6	6.5±0.6		17.3±0.4	45.8±0.6		$Cu_{8.8}Fe_{1.9}As_{5.0}S_{13.3}$
py		32.1±0.3			67.9±0.4		$Fe_{1.0}S_{2.0}$
ccp	23.0±0.6	24.0±0.2			53.0±0.6		$Cu_{0.9}Fe_{1.0}S_{2.1}$
stn	25.0±2.3	12.5±0.8			50.4±0.8	12.2±0.8	$Cu_{2.0}Fe_{1.0}Sn_{1.0}S_{4.0}$

Figure S1 – SEM/BSE images of Neves Corvo raw ore, with phase identification: tennantite (tn), pyrite (py), quartz (qtz), galena (gn), chalcopyrite (ccp) and stannite (stn), and their respective EDS analysis.
